# Circ_0091579 enhances the malignancy of hepatocellular carcinoma via miR-1287/PDK2 axis

**DOI:** 10.1515/biol-2021-0009

**Published:** 2021-01-25

**Authors:** Junwei Shu, Jiayuan Du, Futao Wang, Yong Cheng, Gangxin Chen, Bing Xu, Dianpeng Zhang, Shuangjiang Chen

**Affiliations:** Department of General Surgery, Ankang People’s Hospital of Shanxi Province, Ankang, 6-1-3302, Shifu Courtyard, High-Tech Zone, Ankang 725000, Shanxi Province, China

**Keywords:** hepatocellular carcinoma, circ_0091579, miR-1287, PDK2, glycolysis

## Abstract

Several articles have indicated that circular RNAs are involved in pathogenesis of human cancers. Nevertheless, the role of circ_0091579 in hepatocellular carcinoma (HCC) progression remains to be revealed. Quantitative reverse transcriptase polymerase chain reaction was carried out to examine the expression of circ_0091579 and miR-1287. The proliferation of HCC cells was determined by 3-(4,5-dimethylthiazol-2-yl)-2,5-diphenyltetrazolium bromide (MTT) assay. Flow cytometry was performed to analyze cell cycle progression and apoptosis. Western blot assay was conducted to detect the protein expression of CyclinD1, Cleaved caspase3, and pyruvate dehydrogenase kinase 2 (PDK2). Cell glycolysis was evaluated by measuring the uptake of glucose, the production of lactate, and extracellular acidification rate. The target relationship between miR-1287 and circ_0091579 or PDK2 was verified by dual-luciferase reporter assay, RNA immunoprecipitation assay, and RNA-pull down assay. The enrichment of circ_0091579 was enhanced in HCC tissues (*n* = 77) and four HCC cell lines (HB611, Huh-7, MHCC97, and SNU423) compared with adjacent non-tumor tissues (*n* = 77) and normal human liver cell line THLE-2. Circ_0091579 mediated the promotion of proliferation and glycolysis and the suppression of apoptosis of HCC cells. MiR-1287 was a direct target of circ_0091579 in HCC cells. MiR-1287 knockdown reversed the effects caused by circ_0091579 interference on the functions of HCC cells. PDK2 could bind to miR-1287 in HCC cells. Circ_0091579 upregulated the enrichment of PDK2 by acting as a sponge of miR-1287 in HCC cells. The influence caused by circ_0091579 intervention on HCC cells was attenuated by overexpression of PDK2. Circ_0091579 interference impeded the progression of HCC *in vivo*. Circ_0091579 deteriorated HCC by promoting the proliferation and glycolytic metabolism and suppressing the apoptosis of HCC cells via miR-1287/PDK2 axis.

## Abbreviations

cDNA, complementary DNA
circRNAs, circular RNAs
Cleaved-cas3, Cleaved caspase3
DMEM, Dulbecco’s Modified Eagle Medium
ECAR, extracellular acidification rate
ECL, enhanced chemiluminescent
FITC, fluorescein isothiocyanate
GAPDH, glyceraldehyde-3-phosphate dehydrogenase
HCC, hepatocellular carcinoma
miRNA, microRNA
MTT, 3-(4,5-dimethylthiazol-2-yl)-2,5-diphenyltetrazolium bromide
NSCLC, non-small cell lung cancer
PDK2, pyruvate dehydrogenase kinase 2
PI, propidine iodide
qRT-PCR, quantitative reverse transcriptase polymerase chain reaction
RIP, RNA immunoprecipitation
TNBC, triple negative breast cancer

## 1 Introduction

Hepatocellular carcinoma (HCC) is the fifth prevalent malignant cancer, and also the third leading cause of cancer-related death worldwide [1]. Although surgery and liver transplantation for HCC treatment have been improved, the prognosis of patients with HCC remains dismal because of tumor relapse and metastasis [2,3,4,5]. Therefore, it is urgent to illustrate the potential mechanism behind HCC progression to find better therapeutic strategies for HCC.

Circular RNAs (circRNAs) are featured by covalently closed loop structure without 3ʹ- or 5ʹ-end [6,7]. Many articles reported that circRNAs could function as microRNA (miRNA) sponges [8,9]. The dysregulation of circRNAs has been associated with the pathogenesis of human diseases and cancers [10,11,12,13,14]. Zhang et al. reported that circ_0091579 was aberrantly upregulated in HCC, and the abundance of circ_0091579 was negatively related to the prognosis of patients with HCC [15]. However, the specific mechanism by which circ_0091579 accelerated HCC development needs further investigation.

MiRNAs function as oncogenes or tumor suppressors by targeting 60% human coding genes in cancer cells [16,17]. MiR-1287 was dysregulated in many malignancies [18,19,20]. For instance, Lu et al. found that the miR-1287 level was downregulated in HCC cells, and miR-1287 blocked the proliferation and metastasis of HCC cells via targeting PIK3R3 [21]. In the present study, miR-1287 was predicted as an interacted partner of circ_0091579 via bioinformatics database (Circinteractome). The interaction and functional association between miR-1287 and circ_0091579 were then explored in HCC cells.

Pyruvate dehydrogenase kinases (PDKs) act as crucial modulators in the glycolytic metabolism of cancer cells. Liang et al. reported that PDK2 promoted the glycolytic metabolism of colorectal cancer cells, and dichloroacetate elevated the chemosensitivity of colorectal cancer cells via p53/miR-149-3p/PDK2 axis [22]. He et al. claimed that miR-422a suppressed the malignancy and aerobic glycolysis of gastric cancer cells via PDK2 [23]. Yu et al. found that miR-214 hampered the malignancy and cell metabolism of HCC cells by inhibiting PDK2 and PHF6 [24]. PDK2 was predicted as a candidate target of miR-1287 via StarBase bioinformatics database, and the target interaction and functional association between these two molecules were subsequently investigated in HCC cells.

The clinical data suggested that the high expression of circ_0091579 was associated with short survival time and several malignant clinicopathological characteristics in patients with HCC. In this study, we aimed to disclose the working mechanism of circ_0091579 in HCC progression through experiments *in vitro* and *in vivo*.

## 2 Materials and methods

### 2.1 Patients

In this study, 77 patients with HCC from Ankang People’s Hospital of Shanxi Province were enrolled. The association between clinicopathological characteristics and the expression of circ_0091579 is presented in Table 1.

**Table 1:** Correlation between clinicopathological characteristics and circ_0091579 expression level in patients with HCC

Characteristics	*n*	circ_0091579		*P*
		High (*n* = 39)	Low (*n* = 38)	
**Gender**				
Male	31	19	12	0.125
Female	46	20	26	
**Age (years)**				
≥60	50	28	22	0.201
<60	27	11	16	
**Serum AFP (ng/mL)**				
≥200	49	29	20	0.048*
<200	28	10	18	
**Tumor size**				
≥5 cm	42	26	16	0.030*
＜5 cm	35	13	22	
**TNM stage**				
I + II	40	15	25	0.016*
III + IV	37	24	13	
**Histological differentiation**				
Poorly + moderately	51	29	22	0.127
Well	26	10	16	
**Metastasis**				
Yes	29	20	9	0.012*
No	48	19	29	

AFP: alpha fetoprotein, TNM: tumor node metastasis. *P ＜ 0.05.


**Informed consent:** Informed consent was obtained from all the individuals included in this study.
**Ethical approval:** The research related to human use has been complied with all the relevant national regulations and institutional policies and in accordance with the tenets of the Helsinki Declaration and has been approved by the Ethic Committee of Ankang People’s Hospital of Shanxi Province.

### 2.2 Cell culture

Four human HCC cell lines (HB611, Huh-7, MHCC97, and SNU423) and normal human liver cell line THLE-2 were purchased from BeNa Culture Collection (Beijing, China). All cell lines were maintained in high glucose Dulbecco’s Modified Eagle Medium (DMEM; Gibco, Carlsbad, CA, USA) supplemented with 10% fetal bovine serum (Gibco), 100 U/mL penicillin, and 100 μg/mL streptomycin and grown in an humidified incubator containing 5% CO_2_ at 37°C.

### 2.3 RNA isolation and quantitative reverse transcriptase polymerase chain reaction (qRT-PCR)

RNA sample was extracted from HCC tissues and cells using TRIzol reagent (Invitrogen, Carlsbad, CA, USA). Reverse transcription of miR-1287 was conducted using TaqMan MicroRNA Reverse Transcription Kit (Applied Biosystems, Foster City, CA, USA), and the complementary DNA (cDNA) of circ_0091579 was synthesized using PrimeScript reverse transcriptase reagent kit (Takara, Osaka, Japan). Amplification reaction was conducted using SYBR-Green Master Mix (Takara) on Veriti 96-well Thermal Cycler (Applied Biosystems). qRT-PCR reaction started at 95°C for 3 min followed by 38 cycles of 95°C for 30 s, 60°C for 35 s, and 72°C for 30 s. The expression of circ_0091579 and miR-1287 was analyzed using the 2^−ΔΔCt^ method, and U6 and glyceraldehyde-3-phosphate dehydrogenase (GAPDH) were used as controls. The primers obtained from Sangon Biotech (Shanghai, China) were listed as follows: circ_0091579-forward, 5ʹ-TGAGCCAGTGGTCAGTCAAA-3ʹ and circ_0091579-reverse, 5ʹ-GTGGAGTCAGGCTTGGGTAG-3ʹ; miR-1287-forward, 5ʹ-GTGCTGGATCAGTGGTTC-3ʹ and miR-1287-reverse, 5ʹ-GTCCAGTTTTTTTTTTTTTTTGACTC-3ʹ; U6-forward, 5ʹ-CTCGCTTCGGCAGCACA-3ʹ and U6-reverse, 5ʹ-AACGCTTCACGAATTTGCGT-3ʹ; and GAPDH-forward, 5ʹ-AAGGTCGGAGTCAACGGATTT-3ʹ and GAPDH-reverse, 5ʹ-ACCAGAGTTAAAAGCAGCCCTG-3ʹ.

### 2.4 Cell transfection

Small interfering RNA negative control (si-NC; 5ʹ-UAGCUGACCCGUAGAUUGCAAG-3ʹ), siRNA targeting circ_0091579 (si-circ_0091579; 5ʹ-UUUGUCAAUAAUUUGACUGAC-3ʹ), short hairpin RNA-NC (sh-NC), shRNA targeting circ_0091579 (sh-circ_0091579), miR-1287 mimic (miR-1287; 5ʹ-UACUGCGGUAGAUGCGAUAU-3ʹ), miR-NC (5ʹ-ACUGAUUGACGACGUAUUG-3ʹ), miR-1287 inhibitor (anti-miR-1287; 5ʹ-AGUCGGUAGCGCAUGAAGU-3ʹ), anti-NC (5ʹ-UAACGGCAUUGACAACGUAG-3ʹ), PDK2 ectopic expression plasmid (PDK2), and vector were obtained from Genepharma (Shanghai, China). Empty vector (pLCDH-ciR) and circ_0091579 overexpression plasmid (circ_0091579) were purchased from Ribobio (Guangzhou, China). Transfection was carried out with Lipofectamine 3000 (Invitrogen) when HCC cells reached about 80% confluence.

### 2.5 3-(4,5-Dimethylthiazol-2-yl)-2,5-diphenyltetrazolium bromide (MTT) assay

MTT assay was conducted to assess the proliferation ability of HCC cells. Transfected HCC cells were seeded in 96-well plates in triplicate overnight. After incubation for 24, 48, or 72 h, HCC cells were mixed with 20 μL of MTT (5 mg/mL; Invitrogen) for 4 h. The formazan products were dissolved using 150 μL of dimethyl sulfoxide (Invitrogen). The absorbance at 490 nm was detected using a microplate reader (Bio-Rad, Hercules, CA, USA).

### 2.6 Cell cycle analysis by flow cytometry

HCC cells were collected and fixed using ice-cold 70% ethanol at −20°C overnight. HCC cells were incubated with RNase (10 μM) at 37°C for 30 min. Propidine iodide (PI; Sigma, St. Louis, MO, USA) at a concentration of 5 µg/mL was then incubated with HCC cells at 4°C for 1 h. Cell cycle analysis was conducted using FC-500 flow cytometer (Beckman Coulter, Pasadena, CA, USA).

### 2.7 Cell apoptosis analysis by flow cytometry

The percentage of apoptotic HCC cells was analyzed by flow cytometry after transfection for 72 h. HCC cells were suspended in 200 μL of binding buffer that contained 62.5 ng/mL fluorescein isothiocyanate (FITC)-combined Annexin V (R&D systems, Abingdon, UK) and 5 µg/mL PI (R&D systems) to mark the phosphatidylserine and DNA content for 15 min in the dark at room temperature. The apoptotic HCC cells (FITC^+^ and PI^+/−^) were distinguished from viable or necrotic cells using FC-500 flow cytometer (Beckman Coulter).

### 2.8 Western blot assay

Western blot assay was performed to measure protein expression in HCC cells. The protein samples were isolated with radioimmunoprecipitation lysis buffer (Beyotime, Shanghai, China). Proteins (35 μg) were loaded onto 12% sodium dodecyl sulfate polyacrylamide gel electrophoresis (SDS-PAGE) gels, and then transferred to polyvinylidene fluoride membranes (Millipore, Billerica, MA, USA). Five percent non-fat milk was used to block the nonspecific sites on the membranes. The primary antibodies were all purchased from Abcam (Cambridge, MA, USA). The membranes were incubated with the primary antibody against CyclinD1 (ab134175; dilution: 1:8,000), Cleaved caspase 3 (Cleaved-cas3; ab2302; dilution: 1:10,000), PDK2 (ab68164; dilution: 1:8,000), GAPDH (ab181602; dilution: 1:20,000), or β-actin (ab8226; dilution: 1:20,000) at 4°C overnight. The membranes were washed thrice followed by incubation with secondary antibody (ab6789, Abcam) at room temperature for 2 h. The protein images were visualized using the enhanced chemiluminescent (ECL) system (Beyotime).

### 2.9 Glucose uptake and lactate production detection

Transfected HCC cells were cultivated in glucose-free DMEM for 16 h, and the high-glucose DMEM was added to replace the glucose-free DMEM for 24 h. The glucose level of HCC cells was detected with a fluorescence-based glucose assay kit (BioVision, Milpitas, California, USA). Lactate level in the culture medium was examined with a fluorescence-based lactate assay kit (BioVision).

### 2.10 Extracellular acidification rate (ECAR) detection

Seahorse XF 96 Flux Analyzer (Seahorse Bioscience, North Billerica, MA, USA) was used to measure the real-time ECAR with Seahorse XF Glycolysis Stress Test Kit (Seahorse Bioscience). A total of 4 × 10^4^ HCC cells were plated in a Seahorse XF 96 cell culture microplate. After baseline analysis, 10 mM glucose, 10 μM oxidative phosphorylation inhibitor oligomycin, and 50 mM glycolytic inhibitor 2-deoxyglucose were sequentially injected into the wells at the indicated time points. Data were analyzed by Seahorse XF96 Wave software (Seahorse Bioscience), and ECAR measurement was normalized to cell number.

### 2.11 Dual-luciferase reporter assay

Circinteractome and StarBase were used to seek the downstream genes of circ_0091579 and miR-1287. Dual-luciferase reporter assay was conducted to test the interaction between miR-1287 and circ_0091579 or PDK2.

The fragments of circ_0091579, including the putative binding sites (wild-type or mutant type) with miR-1287, were amplified using PCR and inserted to the PYr-MirTarget luciferase vector (Ambion, Austin, TX, USA). HCC cells were seeded into 24-well plates and co-transfected with miR-NC or miR-1287 and circ_0091579 WT or circ_0091579 MUT using Lipofectamine 3000 (Invitrogen). The relative luciferase activity was measured using Dual-Luciferase Reporter Assay (Promega, Madison, WI, USA), and Renilla luminescence served as the internal control.

The 3ʹ-untranslated region (3ʹ-UTR) fragments of PDK2, containing the wild-type or mutant type of predicted binding sites with miR-1287, were cloned into the PYr-MirTarget luciferase vector (Ambion), generating PDK2 WT or PDK2 MUT. The rest of the procedures were same as above.

### 2.12 RNA immunoprecipitation (RIP) assay

The combination between miR-1287 and circ_0091579 was confirmed by RIP assay. HCC cells were disrupted using Tris-HCl buffer (pH 7.5, 25 mM) and RNase inhibitor (100 U/mL, Sigma). Sepharose beads purchased from Bio-Rad were coated with Argonaute-2 antibody (anti-Ago2) or immunoglobulin G antibody (anti-IgG). Cell lysate was then incubated with the above pre-coated sepharose beads for 3 h at 4°C. qRT-PCR was carried out to detect the expression of circ_0091579 and miR-1287 in complexes that were pulled down by sepharose beads.

### 2.13 RNA-pull down assay

RNA-pull down assay was used to confirm the target relationship between circ_0091579 and miR-1287 in HCC cells. Biotin-labeled miR-NC or miR-1287 was named as Bio-miR-NC or Bio-miR-1287. A total of 100 pmol Bio-miR-NC or Bio-miR-1287 was added to incubate with 2 µg cell lysates. Afterward, 100 µL of streptavidin agarose beads (Invitrogen) was added to incubate for 1 h at room temperature. After boiling the beads in SDS buffer, qRT-PCR was performed to determine the level of circ_0091579.

### 2.14 Murine xenograft model

Murine xenograft model was established to explore the role of circ_0091579 in xenograft tumor growth *in vivo*. A total of 10 BALB/c mice (4 weeks old) were purchased from Chinese Academy of Medical Sciences (Beijing, China). A total of 2 × 10^6^ MHCC97 cells in 150 μL of phosphate buffered saline expressing sh-NC or sh-circ_0091579 were subcutaneously injected into the right side of the back of the mice (*n* = 5). The volume of tumors was monitored every week by volume = (width^2^ × length)/2. Mice were killed and the xenograft tumors were dissected and weighed after injection for 4 weeks. The abundance of circ_0091579, miR-1287, and PDK2 protein was measured by qRT-PCR and western blot assay.

### 2.15 Statistical analysis

The data were analyzed using GraphPad Prism 7 and represented as mean ± standard deviation. The survival rate of patients with HCC was assessed by Kaplan–Meier analysis. Comparisons were analyzed using Student’s *t*-test and one-way analysis of variance followed by Tukey’s test. *χ* ^2^ test was used to evaluate the correlation between the enrichment of circ_0091579 and clinicopathological features of patients with HCC. The linear relationship was assessed by Spearman’s coefficient. *P* < 0.05 indicated a significant difference.

## 3 Results

### 3.1 Circ_0091579 is highly expressed in HCC

**Figure 1:**
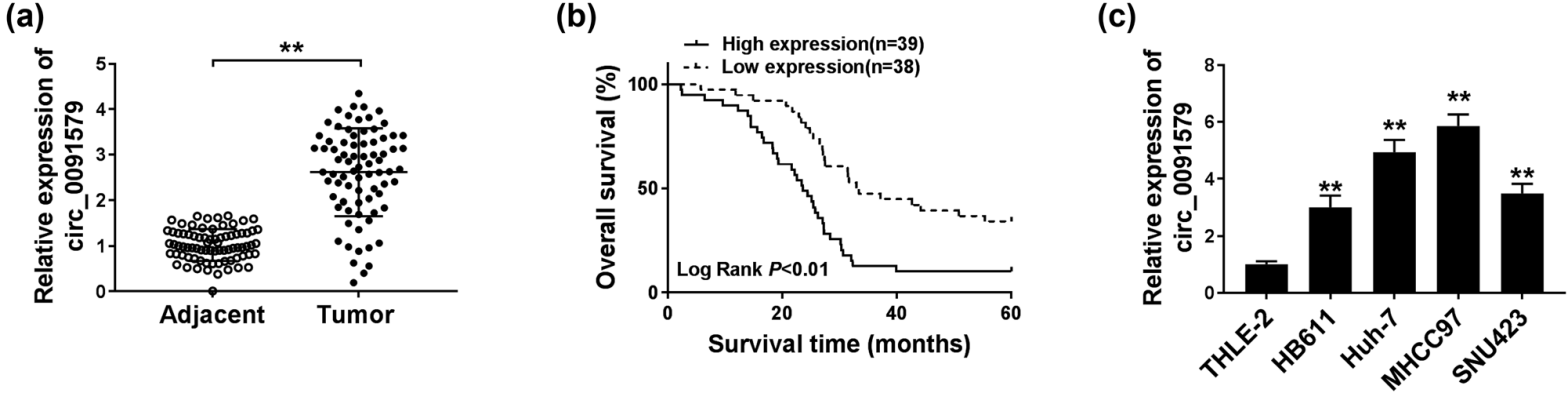
Circ_0091579 is highly expressed in HCC. (a) The expression of circ_0091579 was detected in HCC tissues and adjacent normal tissues by qRT-PCR. (b) The overall survival of patients diagnosed with HCC was analyzed by Kaplan–Meier analysis. (c) qRT-PCR was performed to measure the expression of circ_0091579 in four HCC cell lines and normal human liver cell line THLE-2. **P < 0.01.

The expression of circ_0091579 in HCC tissues (*n* = 77) and paired non-tumor tissues (*n* = 77) was detected by qRT-PCR. As shown in Figure 1a, the level of circ_0091579 was significantly increased in HCC tumor tissues compared with that in adjacent normal tissues. Patients with HCC were divided into high expression group (*n* = 39) and low expression group (*n* = 38) according to the median value of circ_0091579 expression. Patients with HCC with high expression of circ_0091579 were associated with a low overall survival (Figure 1b). Furthermore, we assessed the association between circ_0091579 level and clinicopathological characteristics in patients with HCC. As shown in Table 1, circ_0091579 expression was positively correlated with the concentration of serum alpha fetoprotein, tumor size, tumor node metastasis stage, and metastasis in patients with HCC. A panel of HCC cells, including HB611, Huh-7, MHCC97, and SNU423, along with normal human liver cells THLE-2 was used to detect the expression of circ_0091579. As mentioned in Figure 1c, the level of circ_0091579 was markedly upregulated in HCC cells than that in THLE-2 cells. Taken together, circ_0091579 might serve as an oncogene in HCC.

### 3.2 Circ_0091579 promotes proliferation and glycolysis and suppresses apoptosis in HCC cells

**Figure 2:**
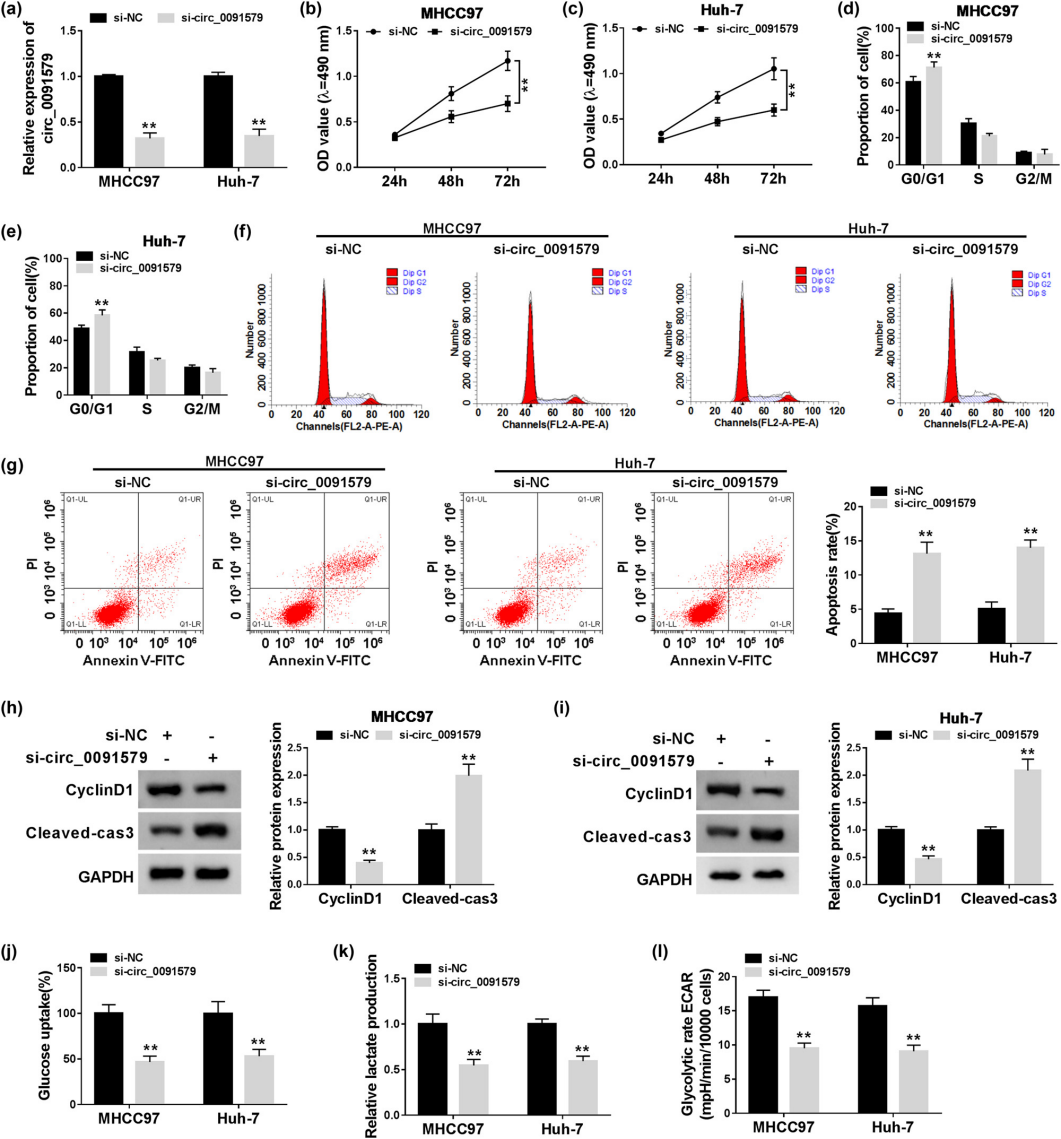
Circ_0091579 promotes the proliferation and glycolysis and suppresses the apoptosis in HCC cells. MHCC97 and Huh-7 cells were transfected with si-NC or si-circ_0091579. (a) The abundance of circ_0091579 was determined in HCC cells by qRT-PCR. (b and c) The proliferation of HCC cells was examined by MTT assay. Flow cytometry was conducted to analyze (d–f) cell cycle progression and (g) the apoptosis of HCC cells. (h and i) Western blot assay was performed to measure the expression of CyclinD1 and Cleaved-cas3 in HCC cells. (j and k) The relative rates of glucose uptake and lactate production were analyzed using fluorescence-based glucose and lactate assay kits. (l) The ECAR of HCC cells was analyzed using Seahorse XF24 Flux Analyzer. **P < 0.01.

**Figure A1:**
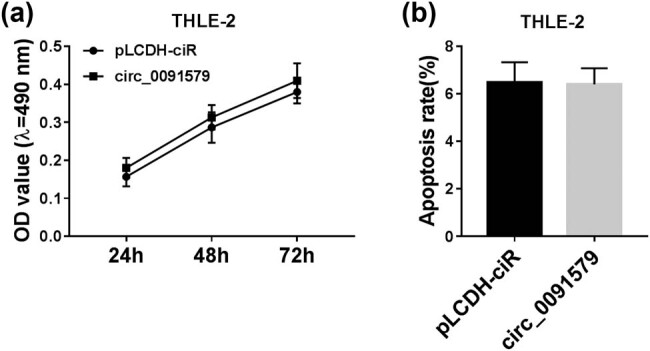
Circ_0091579 overexpression has no influence on the proliferation and apoptosis of THLE-2 cells. THLE-2 cells were transfected with pLCDH-ciR empty vector or circ_0091579 plasmid. (a) MTT assay was performed to generate cell proliferation curve by analyzing the number of THLE-2 cells after transfection for 24, 48, or 72 h. (b) Flow cytometry was conducted to evaluate the apoptosis rate in transfected THLE-2 cells.

HCC cells were transfected with si-NC or si-circ_0091579 to conduct loss-of-function experiments. A notable decrease in circ_0091579 was observed in MHCC97 and Huh-7 cells transfected with si-circ_0091579 (Figure 2a), suggesting the high transfection efficiency of si-circ_0091579 in HCC cells. Circ_0091579 interference inhibited the proliferation of HCC cells (Figure 2b and c). With the interference of circ_0091579, the percentage of HCC cells in G0/G1 was elevated, whereas the percentage of HCC cells in the S phase was reduced (Figure 2d–f), suggesting that circ_0091579 interference arrested cell cycle progression of HCC cells at G1/S transition. The apoptosis of HCC cells was significantly promoted in the si-circ_0091579 group compared with that in the si-NC group (Figure 2g). To further confirm the influences of circ_0091579 silencing on the proliferation and apoptosis of HCC cells, we measured the expression of CyclinD1 and Cleaved-cas3 in HCC cells transfected with si-NC or si-circ_0091579. As shown in Figure 2h and i, with the interference of circ_0091579 in HCC cells, CyclinD1 was notably downregulated, whereas Cleaved-cas3 was upregulated. Furthermore, we detected the glycolysis of HCC cells by measuring the glucose uptake, lactate production, and ECAR. The interference of circ_0091579 decreased the glucose uptake, lactate production, and ECAR of HCC cells (Figure 2j–l), showing that circ_0091579 silencing restrained the glycolytic metabolism of HCC cells. We also assessed the effects of circ_0091579 overexpression on the proliferation and apoptosis of normal human liver cell line THLE-2 to test if circ_0091579 overexpression endowed normal THLE-2 cells the properties of cancer cells. As shown in Appendix Figure A1a and b, the proliferation ability and apoptosis rate of THLE-2 cells were similar in the pLCDH-ciR group and the circ_0091579 overexpression group, suggesting that circ_0091579 overexpression had no influence on the behavior of normal liver cell line THLE-2. These findings showed that circ_0091579 promoted the progression of HCC *in vitro*.

### 3.3 MiR-1287 is a specific target of circ_0091579 in HCC cells

**Figure 3:**
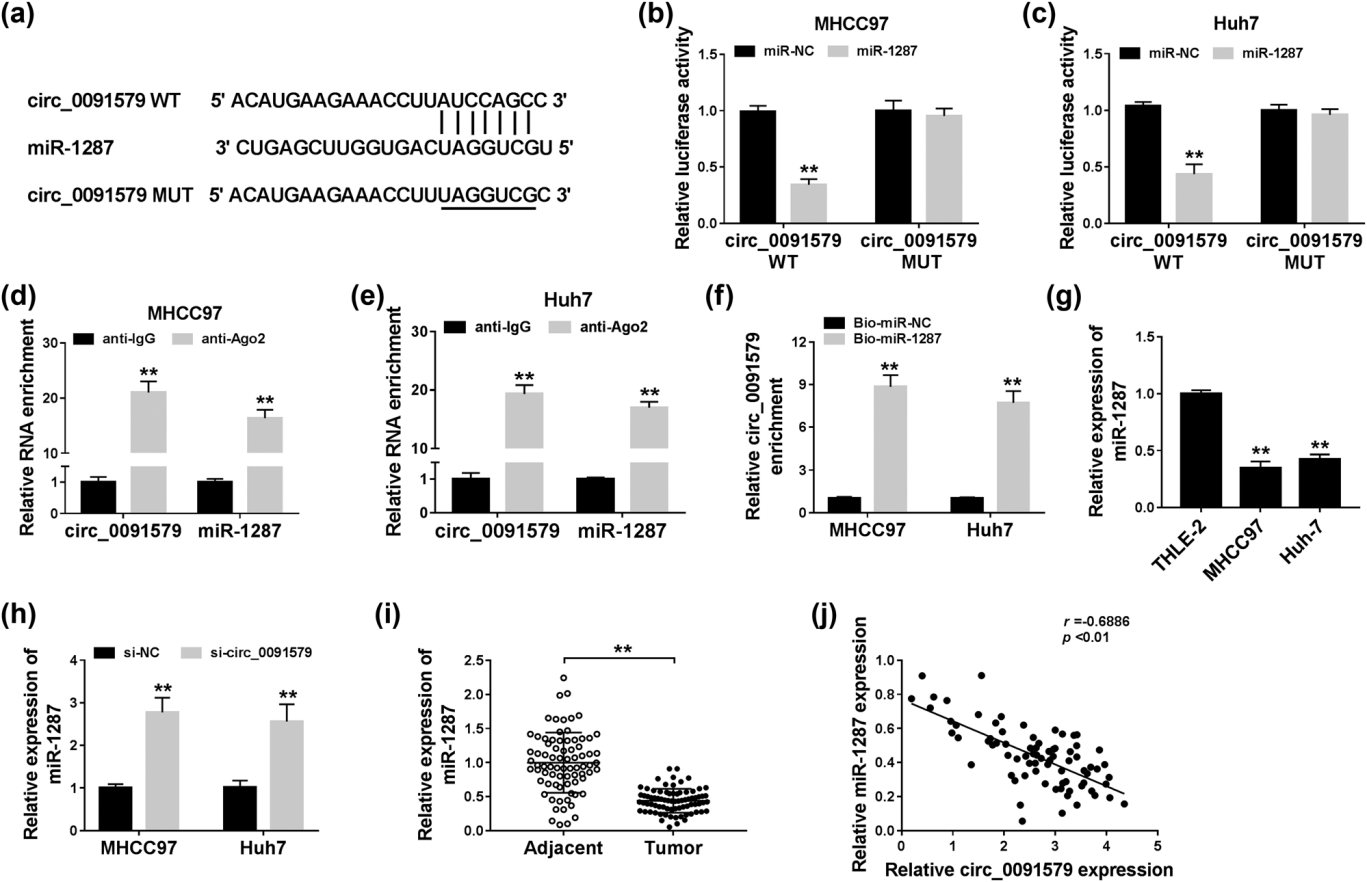
MiR-1287 is a specific target of circ_0091579 in HCC cells. (a) The predicted binding sequence and corresponding mutant sequence of miR-1287 in circ_0091579 were shown as a diagram. (b and c) Dual-luciferase reporter assay was carried out to confirm the interaction between circ_0091579 and miR-1287 in MHCC97 and Huh-7 cells. (d and e) The target relationship between circ_0091579 and miR-1287 was validated in the two HCC cells by RIP assay. (f) RNA-pull down assay was carried out to verify the combination between circ_0091579 and miR-1287 in HCC cells. (g) The abundance of miR-1287 was measured in HCC cells (MHCC97 and Huh-7) and THLE-2 cells by qRT-PCR. (h) The expression of miR-1287 was examined in HCC cells transfected with si-NC or si-circ_0091579 by qRT-PCR. (i) The enrichment of miR-1287 was examined in HCC tissues and adjacent non-tumor tissues by qRT-PCR. (j) The linear relationship between miR-1287 and circ_0091579 was analyzed by Spearman’s correlation coefficient. **P < 0.01.

MiR-1287 was predicted as a target of circ_0091579 by Circinteractome database (Figure 3a). Dual-luciferase reporter assay showed that the luciferase activity was markedly reduced in miR-1287 and circ_0091579 WT co-transfected groups other than miR-NC and circ_0091579 WT groups (Figure 3b and c), suggesting the interaction between miR-1287 and circ_0091579 in HCC cells. RIP assay revealed that circ_0091579 and miR-1287 were both immunoprecipitated when using Ago2 antibody, suggesting that circ_0091579 could bind to RNA-induced silencing complex, likely through the combination with miR-1287 (Figure 3d and e). The level of circ_0091579 was high using Bio-miR-1287 compared with that in the Bio-miR-NC group (Figure 3f), which further confirmed the target interaction between circ_0091579 and miR-1287 in HCC cells. The level of miR-1287 was lower in HCC cells than that in THLE-2 cells (Figure 3g). Si-circ_0091579 transfection caused a notable increase in the expression of miR-1287 in HCC cells (Figure 3h). Besides, the enrichment of miR-1287 was downregulated in HCC tissues (*n* = 77) in comparison with that in adjacent normal tissues (*n* = 77) (Figure 3i). Correlation analysis showed that the expression of circ_0091579 was negatively correlated with the expression of miR-1287 in HCC tissues (Figure 3j). Collectively, miR-1287 could bind to circ_0091579, and it was negatively modulated by circ_0091579 in HCC cells.

### 3.4 Circ_0091579 plays an oncogenic role by directly suppressing miR-1287 in HCC

**Figure 4:**
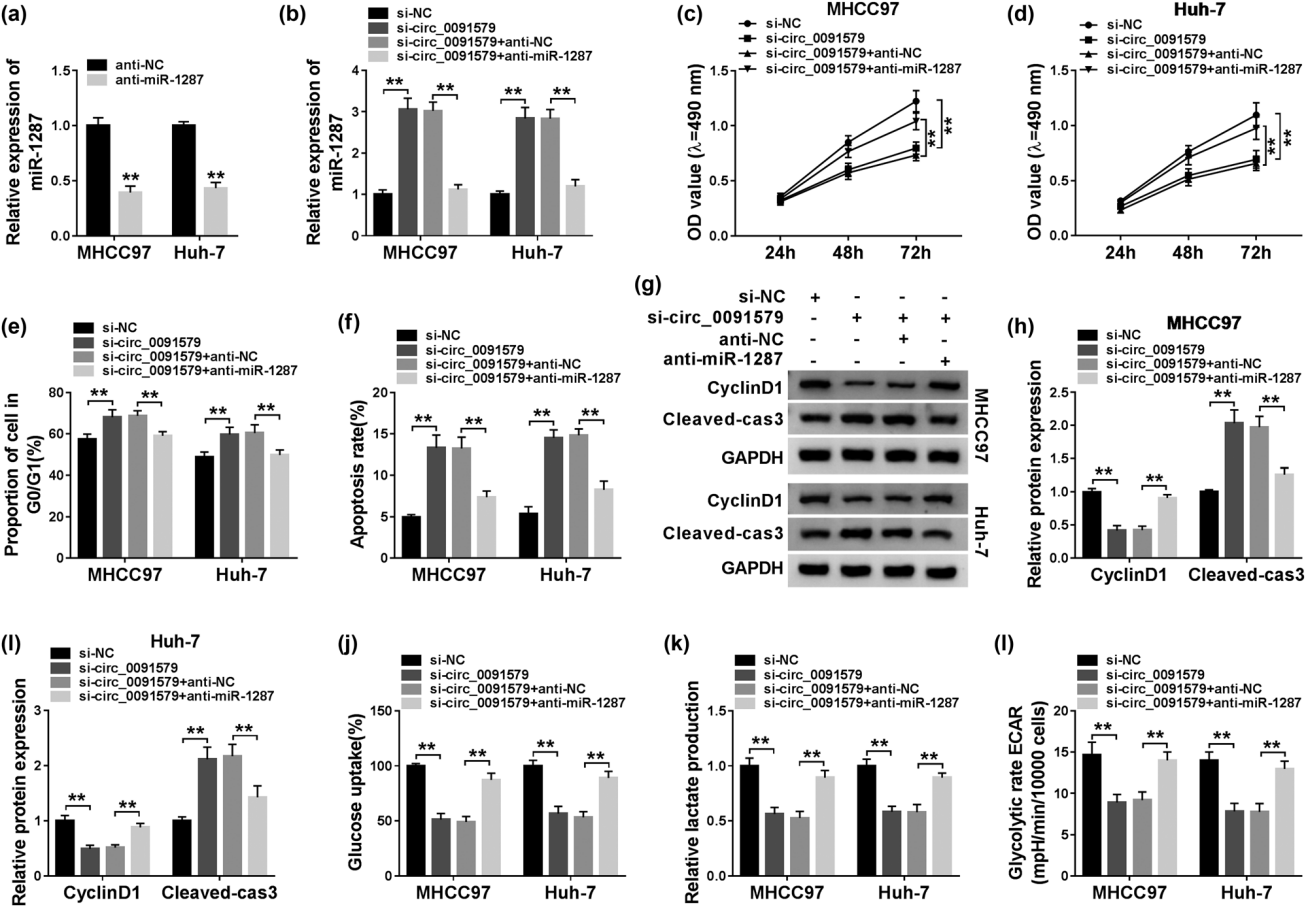
Circ_0091579 plays an oncogenic role by directly suppressing miR-1287 in HCC. (a) The interference efficiency of anti-miR-1287 was assessed in HCC cells by qRT-PCR. (b–l) si-NC, si-circ_0091579, si-circ_0091579 + anti-NC, or si-circ_0091579 + anti-miR-1287 were transfected into HCC cells. (b) The expression of miR-1287 was determined by qRT-PCR. (c and d) MTT assay was performed to measure the proliferation of HCC cells. (e and f) The cell cycle and apoptosis of HCC cells were determined by conducting flow cytometry. (g–i) The protein expression of CyclinD1 and Cleaved-cas3 was examined in HCC cells by western blot assay. (j and k) Fluorescence-based glucose and lactate assay kits were used to evaluate the glucose consumption and lactate production of HCC cells. (l) The glycolytic rate ECAR was detected using Seahorse XF24 Flux Analyzer. **P < 0.01.

The abundance of miR-1287 was reduced in anti-miR-1287 transfected HCC cells compared with that in anti-NC transfected HCC cells (Figure 4a), suggesting that the silencing efficiency of anti-miR-1287 was high in HCC cells. Given the results that circ_0091579 negatively regulated miR-1287 expression, we conducted rescue experiments by transfecting si-circ_0091579 alone or together with anti-miR-1287 into HCC cells. Circ_0091579 interference upregulated the level of miR-1287 in HCC cells, and the addition of anti-miR-1287 reduced the expression of miR-1287 again (Figure 4b). As indicated in Figure 4c–e, circ_0091579 silencing suppressed the proliferation and cell cycle progression of HCC cells, and the transfection with anti-miR-1287 recovered the proliferation and cell cycle progression of HCC cells. The apoptosis of HCC cells was promoted with the intervention of circ_0091579, and the addition of anti-miR-1287 decreased the apoptosis rate of HCC cells (Figure 4f). Western blot assay also showed that the expression of CyclinD1 in HCC cells was restrained by the transfection of si-circ_0091579; the co-transfection of si-circ_0091579 and anti-miR-1287 regained the expression of CyclinD1, and the abundance of Cleaved-cas3 revealed an opposite trend to CyclinD1 (Figure 4g–i). The interference of circ_0091579 inhibited the glycolytic metabolism, and the addition of anti-miR-1287 recovered the glycolysis of HCC cells (Figure 4j–l). In summary, circ_0091579 promoted the development of HCC via sponging miR-1287.

### 3.5 MiR-1287 directly targets and negatively regulates PDK2 in HCC cells

**Figure 5:**
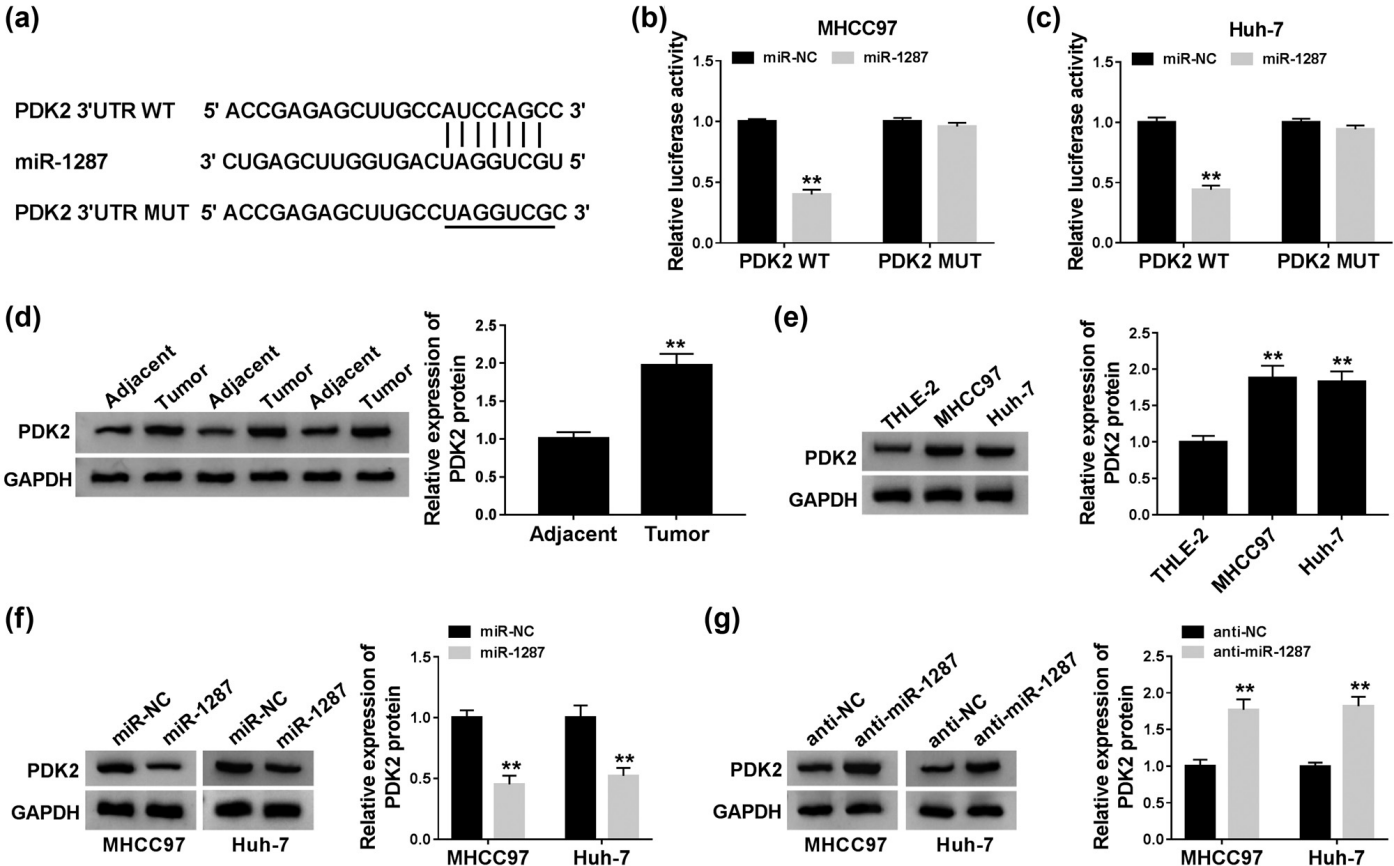
MiR-1287 directly targets and negatively regulates PDK2 in HCC cells. (a) StarBase software predicted that PDK2 was a direct target of miR-1287. (b and c) The combination between miR-1287 and PDK2 was verified by dual-luciferase reporter assay. (d and e) Western blot was conducted to measure the protein expression of PDK2 in HCC tissues and cells. (f) The expression of PDK2 was detected in HCC cells transfected with miR-NC or miR-1287 by western blot assay. (g) Western blot assay was carried out to detect the expression of PDK2 in HCC cells transfected with anti-NC or anti-miR-1287. **P < 0.01.

To illustrate the working mechanism of miR-1287 in the progression of HCC, StarBase database was used to search the targets of miR-1287. As shown in Figure 5a, the 3ʹ-UTR of PDK2 possessed the complementary sites with miR-1287. The overexpression of miR-1287 in the PDK2 WT group notably decreased the luciferase activity compared with the miR-NC group and the PDK2 WT group, and the transfection of miR-1287 or miR-NC in the PDK2 MUT group had no influence on the luciferase activity (Figure 5b and c), suggesting the interaction between miR-1287 and PDK2 in HCC cells. PDK2 was highly expressed in HCC tumor tissues relative to matching normal tissues (Figure 5d). The abundance of PDK2 was also upregulated in MHCC97 and Huh-7 cells compared with THLE-2 cells (Figure 5e). We transferred miR-1287 and anti-miR-1287 into HCC cells to measure the expression of PDK2 under miR-1287 overexpression and interference conditions. As mentioned in Figure 5f and g, miR-1287 accumulation decreased the protein expression of PDK2 in the two HCC cells, and PDK2 was upregulated with the intervention of miR-1287 in MHCC97 and Huh-7 cells. Taken together, PDK2 was a downstream gene of miR-1287, and PDK2 was negatively regulated by miR-1287 in HCC cells.

### 3.6 PDK2 is regulated by circ_0091579/miR-1287 axis in HCC cells

**Figure 6:**
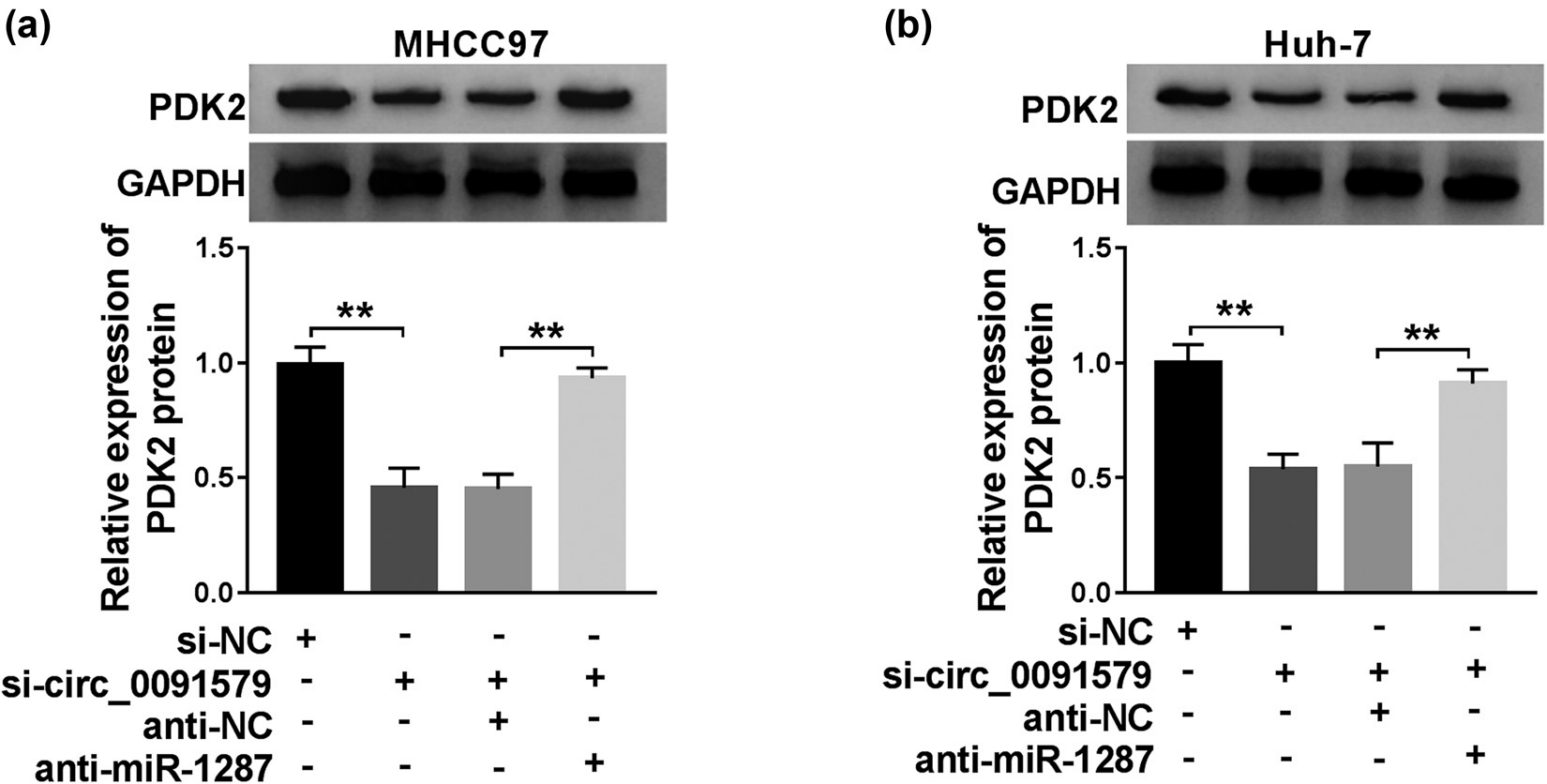
PDK2 is regulated by circ_0091579/miR-1287 axis in HCC cells. (a and b) The protein level of PDK2 was examined in HCC cells transfected with si-NC, si-circ_0091579, si-circ_0091579 + anti-NC, or si-circ_0091579 + anti-miR-1287 by western blot assay. **P < 0.01.

The regulatory relationship among circ_0091579, miR-1287, and PDK2 was explored by transfecting si-circ_0091579 alone or co-transfecting si-circ_0091579 and anti-miR-1287 into HCC cells. As exhibited in Figure 6a and b, the protein level of PDK2 was restrained with the interference of circ_0091579, and the protein level of PDK2 was recovered in the si-circ_0091579 and anti-miR-1287 group in MHCC97 and Huh-7 cells. In summary, circ_0091579 upregulated the expression of PDK2 by sponging miR-1287 in HCC cells.

### 3.7 Circ_0091579 promotes the progression of HCC by enhancing PDK2 level *in vitro*

**Figure 7:**
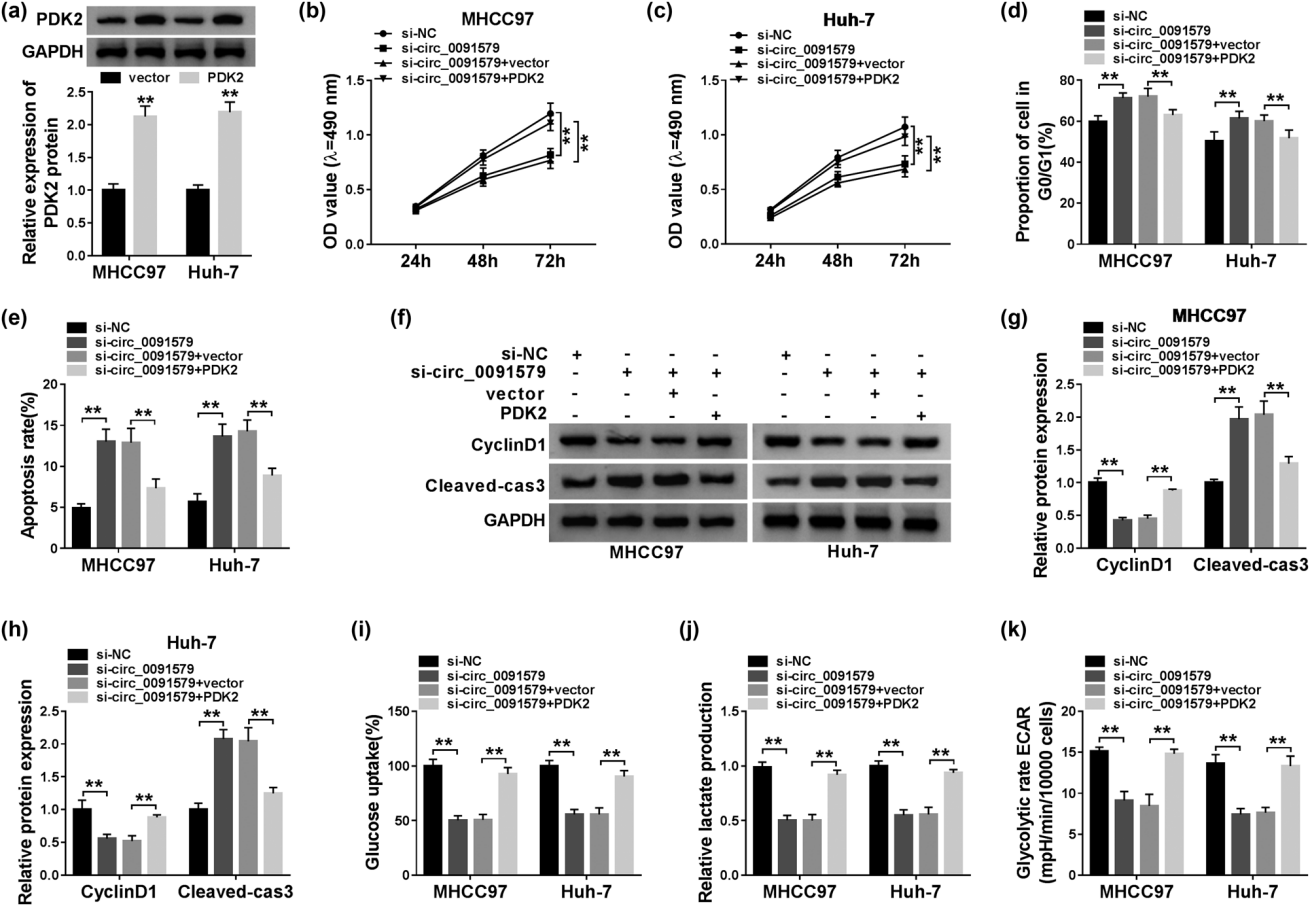
Circ_0091579 promotes the progression of HCC by enhancing PDK2 level *in vitro*. (a) The overexpression efficiency of PDK2 plasmid was evaluated by western blot assay. (b–k) HCC cells were transfected with si-NC, si-circ_0091579, si-circ_0091579 + vector, or si-circ_0091579 + PDK2. (b and c) The optical density at 490 nm of HCC cells was measured by MTT assay. (d and e) Flow cytometry was performed to detect the cycle and apoptosis rate of HCC cells. (f–h) The enrichment of CyclinD1 and Cleaved-cas3 was determined in HCC cells by western blot assay. (i and j) The glucose uptake and lactate production of HCC cells were examined by their corresponding kits. (k) Seahorse XF24 Flux Analyzer was used to assess the ECAR of HCC cells. **P < 0.01.

The overexpression efficiency of PDK2 plasmid was assessed in HCC cells. As exhibited in Figure 7a, the transfection of PDK2 significantly upregulated the expression of PDK2 in MHCC97 and Huh-7 cells. To address the functions of PDK2 on circ_0091579-mediated development of HCC, we conducted rescue experiments. PDK2 overexpression attenuated the inhibitory effects aroused by circ_0091579 interference on the proliferation and cell cycle progression and the promoting effect on the apoptosis of HCC cells (Figure 7b–e). The changes in the abundance of proliferation-associated protein CyclinD1 and apoptosis-related protein Cleaved-cas3 in four groups also supported the above conclusion (Figure 7f–h). The glycolytic analysis showed that PDK2 accumulation overturned the suppressive influence caused by circ_0091579 silencing on the glucose uptake, lactate production, and ECAR of HCC cells (Figure 7i–k). In summary, circ_0091579 exerted its oncogenic role in HCC by upregulating PDK2.

### 3.8 Circ_0091579 intervention blocks the progression of HCC *in vivo*

**Figure 8:**
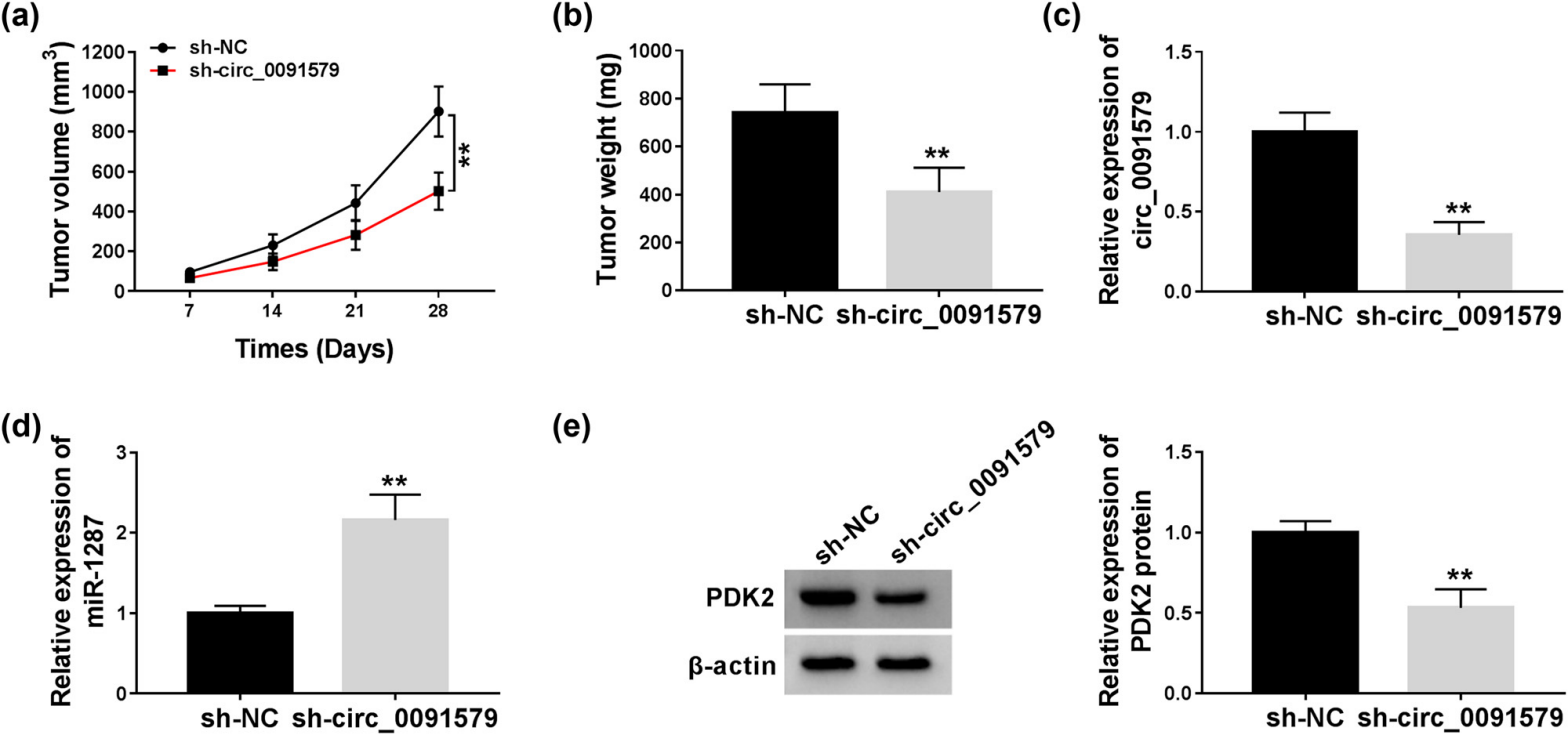
Circ_0091579 intervention blocks the progression of HCC *in vivo*. (a) The tumor volume was measured every week. (b) The tumor weight was detected after injection for 4 weeks. (c–e) The abundance of circ_0091579, miR-1287, and PDK2 protein was measured in HCC tissues by qRT-PCR and western blot. **P < 0.01.

To explore if circ_0091579 exerted the same role *in vivo*, we established a murine xenograft model using MHCC97 cells stably expressing sh-circ_0091579 or sh-NC. As shown in Figure 8a and b, circ_0091579 interference inhibited the tumor growth of HCC, and the dimension and weight of HCC tumors were smaller in the sh-circ_0091579 group than that in the sh-NC group. The abundance of circ_0091579, miR-1287, and PDK2 was detected in HCC tissues dissected from mice in the sh-NC group and the sh-circ_0091579 group. As shown in Figure 8c–e, a significant decrease in the expression of circ_0091579 and PDK2 protein was found in the sh-circ_0091579 group, whereas the interference of circ_0091579 notably increased the expression of miR-1287 in HCC tumor tissues. Collectively, circ_0091579 accelerated the growth of HCC tumors *in vivo*.

## 4 Discussion

CircRNAs play important roles in human cancers. For instance, Yang et al. claimed that circ_0046264 restrained the progression of lung cancer by upregulating BRCA2 via sponging miR-1245 [25]. Zhang et al. found that circ_0072995 facilitated the motility of breast cancer cells by sponging miR-30c-2-3p [26]. Huang et al. reported that circ_0000144 accelerated the development of bladder cancer by miR-217/RUNX2 signaling [27]. In the current study, we focused on the functions of circ_0091579 in HCC cells. The expression of circ_0091579 was prominently elevated in HCC tissues and cell lines relative to adjacent non-tumor tissues and THLE-2 cells. Besides, high expression of circ_0091579 was related to the low overall survival of patients with HCC. These findings implied that circ_0091579 might serve as an oncogene in HCC. To explore the role of circ_0091579 in HCC cells, loss-of-function experiments were conducted. The results revealed that circ_0091579 silencing restrained the proliferation and glycolytic metabolism, and induced the apoptosis of HCC cells.

CircRNAs have been reported to function as miRNA sponges in cancer cells [9,28]. For instance, circ_101505 elevated the chemoresistance of HCC cells to cisplatin by sponging miR-103, thus enhancing the expression of NOR1 [29]. In this study, miR-1287 was confirmed as a target of circ_0091579, and further experiments revealed that miR-1287 was negatively regulated by circ_0091579 in HCC cells. The anti-tumor role of miR-1287 has been reported in HCC, triple negative breast cancer (TNBC), and non-small cell lung cancer (NSCLC) [21,30,31]. For instance, Lu et al. reported that miR-1287 acted as a tumor suppressor in HCC, and it repressed the malignancy of HCC cells by downregulating PIK3R3 [21]. Schwarzenbacher et al. found that miR-1287-5p could inhibit the growth of TNBC [31]. Li et al. proved that circ_0016760 accelerated the progression of NSCLC via miR-1287/GAGE1 signaling [30]. Rescue experiments were carried out to disclose if circ_0091579 functioned by sponging miR-1287 in HCC cells. The inhibitory effects of circ_0091579 knockdown on the malignant behaviors of HCC cells were partly overturned by the addition of anti-miR-1287, showing that circ_0091579 silencing hampered cell proliferation and glycolysis and triggered the apoptosis of HCC cells partly by upregulating the expression of miR-1287.

MiRNAs have been reported to act as important regulators by targeting mRNAs in cancer cells [16,17]. For instance, miR-218 blocked the metastasis of HCC cells by targeting and suppressing SERBP1 [32]. StarBase database was used to search the candidate targets of miR-1287, and we concentrated on PDK2 because of its important role in glycolytic metabolism. Subsequently, dual-luciferase reporter assay confirmed the interaction between miR-1287 and PDK2. There are four members of PDK family, namely PDK1–4 [33–35]. Diverse PDKs might serve different functions in cancer progression. Dupuy et al. found that PDK1 promoted the progression of breast cancer by accelerating the metabolic pathway [36]. Li et al. proved that miR-182 accelerated the tumorigenesis of lung cancer by inhibiting PDK4, suggesting that PDK4 played a tumor suppressor role in lung cancer [37]. PDK2 was identified as an oncogene in HCC by previous studies. For instance, miR-214 restrained HCC progression by suppressing PDK2 and PHF6 [24]. Wei et al. reported that DUXAP8 accelerated HCC development by upregulating PDK2 via sequestering miR-422a [38]. Herein, we found that PDK2 was modulated by circ_0091579/miR-1287 axis in HCC cells, and circ_0091579 played an oncogenic role in HCC by upregulating PDK2.

The *in vivo* role of circ_0091579 was also verified by establishing the murine xenograft model. The results of murine xenograft model showed that circ_0091579 interference impeded the growth of HCC tumors *in vivo*.

In summary, circ_0091579 potentiated the proliferation and glycolysis, whereas it prevented the apoptosis of HCC cells by upregulating PDK2 via serving as a sponge for miR-1287. Circ_0091579/miR-1287/PDK2 axis is identified in this study for the first time, and this might provide new insight to find an effective treatment strategy for patients with HCC.
